# A methodological framework for validating a multi-domain physiological sensor for divers using a scalable data fusion platform

**DOI:** 10.3389/fphys.2026.1759280

**Published:** 2026-03-18

**Authors:** Paul Beatty, Marshall Tumperi, Harrison Nguyen, Danielle Howe, Paul Hage, Robert Wilson, Bryndan Lindsey, Jade Kandel, Brett Collar, Sara Gravelyn, Gregory Fulmer, Kinjal Sethuraman, Austin Veith

**Affiliations:** 1 The Johns Hopkins University Applied Physics Laboratory, Laurel, MD, United States; 2 Department of Computer Science, The University of North Carolina at Chapel Hill, Chapel Hill, NC, United States; 3 University of Maryland Medical Center – Shock Trauma, Baltimore, MD, United States

**Keywords:** data fusion, diver physiological monitoring, human performance, ROS 2, wearables

## Abstract

Divers face numerous physiological hazards that can lead to diving disorders, yet the underlying mechanisms remain poorly understood due to limitations in monitoring technologies capable of functioning underwater. To bridge this gap, it is necessary to critically evaluate novel monitoring technologies and develop approaches to determine their suitability for near real-time physiological assessment. Gold standard devices are typically used to validate new physiological sensors under controlled normobaric conditions, where their accuracy benefits from stable, low-interference environments. However, the absence of underwater gold standards and the complexity of underwater conditions make it difficult to determine whether measurement discrepancies arise from true physiological changes or sensor variability. Given the wide range of physiological responses elicited by diving, it is essential to deconstruct the underwater experience into its constituent factors to better isolate their individual effects. This article presents a comprehensive methodology for evaluating the performance of a novel physiological sensor designed for both land-based and underwater environments. To this end, human subject testing was conducted across three experimental environments: 1. dry normobaric testing, 2. dry hyperbaric testing, and 3. shallow water immersion. To facilitate these evaluations, a custom data acquisition platform was developed on Robot Operating System 2 (ROS 2), enabling coordinated synchronization of multiple heterogeneous data streams. This approach offers a scalable and reproducible framework for validating physiological monitoring technologies.

## 1 Introduction

Understanding human performance and physiological response in underwater environments is critical to ensuring diver safety. Dive-related disorders can manifest at any phase of a dive (descent, bottom time, ascent, or post-dive) and result from a variety of external factors. These conditions vary in pathophysiological mechanisms and clinical severity, ranging from mild discomfort to permanent injury to death (Beatty et al., 2024; Naval Sea Systems Command, 2016). For example, barotrauma (e.g., ear squeeze (Mawle and Jackson, 2002) or subcutaneous emphysema (Aghajanzadeh et al., 2015)) most commonly occurs during ascent or descent, caused by pressure differences across gas-filled spaces within the body (Hamilton-Farrell and Bhattacharyya, 2004), and can develop even at relatively shallow depths if pressure equalization is insufficient (Sheridan et al., 1999). During bottom time, disorders predominantly arise from the composition and partial pressures of inspired gases (e.g., hypoxia (Wiebe et al., 1999), oxygen toxicity (van Ooij et al., 2013), hypercapnia (Dunworth et al., 2017), or nitrogen narcosis (Rocco et al., 2019)), while prolonged exposure to environmental factors introduces additional physiological risks (e.g., hyperthermia (Looney et al., 2019), hypothermia (Aguilella-Arzo et al., 2003), or immersion pulmonary edema (Harper, 2024; Wilmshurst et al., 1989)). During ascent, rapid reductions in ambient pressure can cause inert gases to form bubbles within tissues or blood (Vann et al., 2011; Mitchell et al., 2022), which leads to decompression illnesses (e.g., arterial gas embolism, venous gas embolism, or decompression sickness (Neuman, 2002)). While strict adherence to established dive protocols can mitigate many operational risks (Naval Sea Systems Command, 2016), there remains a critical need for underwater sensing technologies capable of providing real-time assessment and early warning of diver health events. However, few such systems currently exist (Beatty et al., 2024).

Commonly documented physiological metrics such as heart rate (HR), pulse rate (PR), respiratory rate (RR), oxygen saturation (SpO_2_), and core body temperature, offer critical insights into a diver’s physiological status, enabling the early detection of emerging disorders (Beatty et al., 2024). Commercial off-the-shelf (COTS) and medical-grade devices feature sensors that are capable of measuring these biosignals (Merrigan et al., 2023; Lee et al., 2014; Murdock and Hagen, 2018; Seshadri et al., 2019; Li et al., 2016). When evaluating new sensors, gold standard devices serve as the reference for ground truth comparison. While these gold standards are well established and validated under controlled normobaric conditions, their performance benefits from stable environments with minimal external interference (Lu et al., 1997; Hripcsak and Wilcox, 2002). The transition to underwater environments introduces substantial challenges in assessing the accuracy and reliability of devices used to measure these physiological parameters. First, the physiological stresses of immersion have the potential to modify expected physiological responses in the diver (Beatty et al., 2024; Looney et al., 2019; Flook, 1987; Levett and Millar, 2008; Mallen and Roberts, 2019; Breskovic et al., 2011; Pendergast et al., 2025). Second, existing land-based gold standard devices largely cannot be submerged in water, hindering direct comparison of surface measurements with those obtained under the dynamic ambient pressures experienced at depth. A key example of this is the difficulties faced when translating ECG devices to saltwater environments (Reyes et al., 2014; Thap et al., 2016). Therefore, even if it were possible to experimentally compare physiological signals measured underwater to those obtained at the surface and quantify their discrepancies, it would remain ambiguous whether these discrepancies originate from genuine physiological alterations or from sensor performance variability and calibration issues in the underwater environment. Beyond the limitations caused by water, variation in data management and formatting strategies across manufacturers and heterogeneous sampling rates across sensors complicates the interpretation and comparison of physiological data collected by disparate devices during human subjects testing (Celik and Godfrey, 2023). A near real-time, sensor-agnostic, time-synchronized data acquisition system could overcome these limitations, streamlining device integration and data analysis (Jo et al., 2021). Such a system would allow for the flexible integration of diverse physiological and environmental sensors, regardless of manufacturer or communication protocol, while maintaining precise temporal alignment across all data streams.

This article describes a multi-phase test and evaluation paradigm to assess the performance of a novel physiological sensor (nPS) developed for dive applications alongside several reference COTS devices. The nPS is a proprietary, third-party, prototype device evaluated by the Johns Hopkins University Applied Physics Laboratory (JHU/APL) under specific non-disclosure constraints. By systematically comparing device agreement with reference instruments and reliability in simulated operational environments, this protocol aimed to determine the nPS’s suitability for supporting underwater physiological monitoring and early detection of health risks. Given that diving elicits a wide range of physiological responses (Pendergast et al., 2025), this protocol broke down the underwater experience into four primary factors—water immersion, temperature, pressure, and breathing gas mixture—to isolate their effects on both human physiology and sensor performance. Immersion in thermoneutral water produces only mild physiological effects (Flux et al., 2022) but can significantly degrade the accuracy of physiological sensors (Reyes et al., 2014; Gradl et al., 2017; Schuster et al., 2017; Röttgers et al., 2014). Modest deviations in water temperature can disrupt thermoregulation (Guo et al., 2024; Kha et al., 2015; Leffler, 2001), though their impact on sensor performance appears minimal (Lasance et al., 1994). Pressure profoundly impacts autonomic regulation of cardiorespiratory function (Hernando et al., 2019) but poses little challenges to most sensors, which are engineered to function reliably across a range of pressures (Lafay et al., 2008; Shen et al., 2018). Finally, even small deviations in breathing gas composition can produce significant physiological responses, especially at depth, where increased pressure alters gas absorption dynamics (Pastena et al., 2005; Eynan et al., 2020; Posada-Quintero et al., 2021), though such variations are unlikely to influence sensor function differently than under normobaric surface conditions.

The testing phases described in this study were structured to isolate and evaluate each environmental factor’s impact on the device’s agreement with reference instruments. The nPS was first assessed in a dry, normobaric environment to establish a baseline device agreement in a control condition. Further tests were conducted under discrete environmental conditions to isolate the effects of pressure and water: dry hyperbaric testing and shallow water immersion. Dry hyperbaric testing evaluated device behavior under elevated ambient pressure in the absence of water, while also allowing for the controlled introduction of alternative breathing gas mixtures in a clinical setting. In contrast, shallow water immersion assessed device behavior while submerged without significant pressure, offering insight into the effects of thermoneutral freshwater exposure in controlled but operationally relevant conditions. Taken together, these tests enabled a comprehensive characterization of device performance, thereby providing insight into future behavior when the device encounters multiple environmental factors simultaneously.

## 2 Materials and equipment

### 2.1 Test locations

Each phase of testing was conducted at a different facility to best accommodate the specific environmental conditions required for the devices under test. Dry normobaric testing was conducted at JHU/APL main campus in Laurel, Maryland. Dry, hyperbaric testing took place at the Center for Hyperbaric Medicine at the University of Maryland Medical Center (UMMC), utilizing the hyperbaric chamber housed within the UMMC R. Adams Cowley Shock Trauma Center in Baltimore, Maryland. Water immersion testing took place at the Neutral Buoyancy Research Facility (NBRF) at the University of Maryland, College Park, which houses a large, 25-foot (7.6 m) dive tank.

### 2.2 Devices under evaluation

In lieu of gold standard, clinical-grade measurements, the nPS was evaluated by comparing each reported physiological metric against equivalent measurements from established commercial and research grade physiological monitors (Table 1). The nPS recorded electrocardiography (ECG), photoplethysmography (PPG), near infrared spectroscopy (NIRS), and derived metrics including SpO_2_ and tissue oxygen saturation (StO_2_). As stated above, the nPS is a proprietary, third-party developed prototype device that was evaluated by JHU/APL under a non-disclosure agreement. Consequently, specific details on the nPS hardware and algorithms have been withheld. Features extracted from the ECG and PPG signals (HR and PR, respectively) were compared with data collected using the BIOPAC (BIOPAC Systems Inc., Goleta, CA, USA) MP160 system with Bionomadix modules (RSPEC-R and PPGED-R), a Nonin Model 3230 Bluetooth® Low Energy fingertip pulse oximeter (Nonin Medical Inc., Plymouth, MN, USA), a Garmin Fenix 8 dive watch (Garmin International, Olathe, KS, USA), and a Polar H10 chest strap (Polar Electro Oy, Kempele, Finland). SpO_2_ measurements were compared with those from the BIOPAC MP160 with the OXY100 module, Nonin Model 3230 Bluetooth® Low Energy and Garmin Fenix 8. StO_2_ measurements were compared with the Moxy Muscle Oxygen Monitor (Fortiori Design LLC, Hutchinson, MN, USA). Skin temperature measurements were compared with the CORE body temperature monitor (Greenteg AG, Zürich, Switzerland).

**TABLE 1:** Devices under evaluation.

#	Type	Sensor	Battery	Form factor	Test scenario	Communication type	Physiological metrics				
							HR	PR	SpO2	StO2	Skin temp††
1	Test Device	nPS	Rechargeable lithium-ion battery	Adhesive	NB, HB, WI	BLE/Local Storage	✓†	✓†	✓	✓	✓
2	Reference Device	BIOPAC Modules* (BIOPAC, 2025)	Rechargeable lithium-ion battery or Wired	Chest Strap/Finger Clip	NB, HB	TCP	✓	✓	✓		
3	COTS Comparison	Nonin Model 3230 Bluetooth® Low Energy (Nonin, 2025)	AAA (dry cell)	Finger clip	HB, WI***	BLE		✓	✓		
4	COTS Comparison	CORE (CORE, 2025)	Rechargeable lithium-polymer battery	Chest strap	NB	BLE					✓
5	COTS Comparison	Garmin Fenix 8 (Garmin, 2025)	Rechargeable lithium-ion battery	Watch	HB, WI	Cloud Storage**		✓	✓		
6	COTS Comparison	Moxy Muscle Oxygen Monitor (Moxy Monitor, 2025)	Rechargeable lithium-polymer battery	Adhesive	NB	BLE				✓	
7	COTS Comparison	Polar H10 (Polar, 2025)	CR 2025 (lithium coin)	Chest strap	NB, HB, WI	BLE/Cloud Storage	✓				

NB, normobaric; HB, hyperbaric; WI, water immersion; BLE, bluetooth low energy; TCP, transmission control protocol; HR, heart rate; PR, pulse rate; SpO_2_, peripheral oxygenation saturation; StO_2_, tissue oxygen saturation; Skin Temp, Skin Temperature.

*BIOPAC modules included the RSPEC-R, PPGED-R, and OXY100.

**The Garmin Fenix 8 was not integrated into the real-time system due to proprietary restrictions.

***Although the Nonin was not submerged as part of water immersion testing (it is not waterproof), it was used during pre/post dive pool-side assessment.

† The nPS collected raw ECG and PPG; Related physiological metrics (e.g., HR, PR) were calculated from the raw signals.

†† Temperature data were recorded, but were not analyzed as part of this study.

The rationale for the device selection is varied, but largely fell into three categories: previously demonstrated hyperbaric resilience, anecdotal popularity among diving populations, and form factor similarity to the nPS. The BIOPAC system and Nonin device are considered accurate reference instruments under hyperbaric conditions, as they have been previously validated in such environments (Hess et al., 2020; Schirato et al., 2020; Ross et al., 2013). Subsequently, the Moxy device was selected for its compatibility with BIOPAC hardware. The Garmin Fenix device was selected due to is dive activity features and integrated physiological sensing. Finally, the Polar device was selected because its form factor is similar to the nPS device, as well as its water proofing.

Not all devices were tested in every scenario. Only waterproof or pressure tolerant devices were included in water immersion or hyperbaric trials, respectively. This selective approach ensured meaningful, scenario-appropriate performance data without unnecessary failure risk.

### 2.3 Data acquisition system

A custom in-house software package, adapted from the approach described by Jo et al. (2021) and paired with a graphical user interface (GUI), was developed to collect and visualize live, time-synchronized data from the sensors in Table 1. The back-end software was built on the open-source middleware Robot Operating System 2 (ROS 2), which provided a standardized node-based architecture and publisher/subscriber interface (Quigley et al., 2009; Macenski et al., 2022). This framework enabled selective recording of device streams and scaling of the system configuration based on the number and type of devices required for each session. To ensure consistent deployment and reproducibility across different computing environments, the software was containerized using Docker, allowing for simplified set up and scalable execution.

**FIGURE 1:**
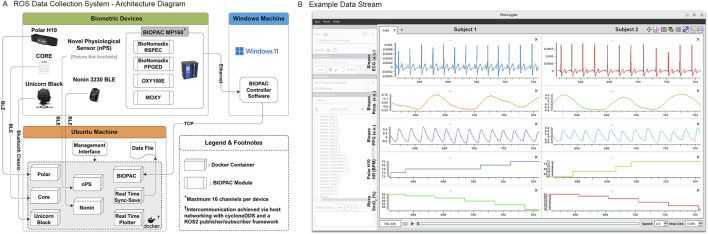
Custom ROS data collection system. **(A)** System architecture implemented using a ROS 2 framework with custom Python scripts deployed via Docker containers. The architecture was scaled according to the number of devices and participants for each collection session. **(B)** PlotJuggler enabled visualization of live ECG, Respiration, PPG, HR, and SmO2 (rows) data from two participants (columns).

As illustrated in Figure 1A, data collection was performed on a Dell laptop (Dell Precision 7780 13th Gen Intel® Core ™ i9-13950HX 64GM RAM) running Ubuntu 24.04 LTS. The software launched one Docker container per device type; each built from a base image configured with Ubuntu 22.04 and ROS 2 Humble. Within each container, a Python-based handler searched for the device via the communication interface specified in Table 1 and Figure 1A, established a connection, initiated data streaming, and published raw data as ROS 2 messages. Feature extraction nodes subscribed to raw data streams (e.g., ECG), computed features (e.g., HR) using Pyphysio (Bizzego et al., 2019), and published the results as ROS 2 messages. Two additional subscriber nodes aggregated all data topics: one synchronized and stored data to an H5 file for offline analysis, and the other enabled real-time visualization via PlotJuggler (Github, 2025) (Figure 1B). PlotJuggler’s customizable, drag-and-drop interface allowed dynamic rearrangement, rescaling, and overlay of plots as the number of session participants, sensor configuration, or computed metrics changed during or between data collection sessions. The system used host networking with Cyclone DDS to enable inter-container communication.

The output H5 file included epoch timestamps with nanosecond precision and columns for each raw and calculated data value. The sensors integrated into the data collection system operated at heterogeneous native sampling rates, reflecting differences in sensing modality, onboard processing, and communication constraints (Table 1). The nPS provided optical signals at approximately 38 Hz and ECG data at 200 Hz, while the BIOPAC reference system sampled all channels at 200 Hz. BIOPAC data were acquired on a Dell laptop (Dell Latitude 5521 11th Gen Intel® Core ™ i7-11850H 16.0 GB RAM) dedicated Windows-based system (Windows 11 Version 23H2 OS Build 22631.5335) and transmitted in real time via a direct Ethernet connection using a TCP/IP interface to the primary Ubuntu-based data collection computer, where the data were injected into the ROS 2 network for synchronization and recording. The Moxy muscle oxygen monitor produced tissue oxygen saturation updates at approximately 0.5 Hz. The Polar H10 chest strap streamed heart rate values in real time at approximately 1 Hz. Consumer wearable devices such as the Nonin Model 3230 fingertip pulse oximeter and the CORE body temperature monitor transmitted processed physiological metrics at approximately 1 Hz. The Garmin Fenix dive watch provided internally processed physiological metrics at device- and activity-dependent intervals without exposing a fixed raw sampling rate for real-time streaming.

To enable unified time-synchronized storage and downstream analysis, a single master sampling rate of 200 Hz was enforced at the synchronization node responsible for generating the session H5 file. This rate was selected because it matched the highest native sampling frequency among the real-time-integrated devices. At the synchronization node, data streams from devices operating below 200 Hz were temporally up-sampled by repeating the most recent valid value until a new sample was received. This placeholder-based approach ensured that each row of the H5 file contained a complete, temporally aligned set of raw and derived data values across all devices. This strategy was implemented solely to simplify data engineering, storage consistency, and real-time visualization; during offline post-processing, device-specific sampling rates were respected and redundant placeholder values were handled appropriately for analysis. In general, if proprietary algorithms were used to calculate heart rate or pulse rate (as is the case for the Garmin device), JHU/APL utilized this value for analysis and no further signal processing was performed.

There were three exceptions to the time-synchronized data collection methodology. First, the Garmin Fenix was not integrated into the real-time system due to proprietary restrictions. For sessions involving the Fenix, data collection was initiated manually on the watch. Data files were retrieved from Garmin’s cloud system post-session and subsequently aligned with the time-synchronized H5 file during offline processing. Second, all devices lost Bluetooth Low Energy (BLE) connection once submerged in water. Consequently, during water immersion testing, data were stored locally onboard each device, retrieved through the manufacturer’s proprietary interface after each session, and aligned during offline processing with the time-synchronized H5 file. Lastly, although the nPS was integrated into the real-time streaming system, final analyses used stored data due to intermittent BLE connectivity. Data were exported from the nPS after each session and aligned, using segments recorded during periods of stable BLE transmission, with the corresponding synchronized dataset in the H5 file.

For devices relying on cached onboard data, including underwater and post-session retrieval cases, temporal alignment was performed using device-generated timestamps. The H5 file employed Python-based system timestamps as a common temporal reference, which were linked to device timestamps during offline processing to establish alignment. For the nPS specifically, both real-time streamed data and cached onboard data were available. A cross-correlation-based alignment procedure was used to reconcile these data sources and to piecewise recover segments affected by intermittent real-time transmission loss using the cached data.

## 3 Methods

### 3.1 Test procedures

Prior to conducting human subjects research, benchtop testing was performed to ensure adherence to safety protocols by pressure-testing all device batteries and verifying the structural integrity of the battery and housing before submerging the devices or placing them inside a hyperbaric chamber. Lithium-ion battery testing standards were reviewed, and benchtop procedures were evaluated by the JHU/APL Environmental Health and Safety (EHS) office to ensure appropriate risk mitigation. A thorough review of safety codes, guidelines, standards, and research publications was performed to ensure compliance for testing each device (see Supplementary Appendix). Additionally, all equipment used in dry hyperbaric testing was approved by the UMMC Technical Advisory Committee (TAC) for use inside the hyperbaric chamber.

All human subjects research was Institutional Review Board (IRB) and Human Research Protection Officer (HRPO)/Office of Human Research Oversight (OHRO) approved. IRB-approved protocols included Johns Hopkins Medicine (JHM) IRB00478482 and IRB00438207, and University of Maryland (UMD) HP-00113146. All procedures performed in studies involving human participants were in accordance with the ethical standards of the institution. Informed consent was obtained from all individual participants included in the study. Participants were recruited independently for each phase of testing, although some individuals participated in both the water immersion and dry hyperbaric phases. A total of 11 participants were recruited for dry normobaric testing, 6 participants (11 dives) for dry hyperbaric testing, and 11 participants (14 dives) for water immersion testing. In the case of the dry hyperbaric dives some participants completed multiple dive profiles. Similarly, three participants completed multiple water immersion dives. Additional participant demographic information is captured in Table 2 below.

**TABLE 2:** Participant demographics.

Environment	Male	Female	Age (mean ± SD)
Dry, normobaric	6	5	28.8 ± 5.4
Dry, hyperbaric	4	2	44.1 ± 14.6
Water immersion	8	3	23.6 ± 1.4

The goal of testing was not to reach significance, but to inform decision-making during device development. Specifically, the focus of the studies described in the following section were to examine the effectiveness of the ROS 2 system while collecting multimodal sensor data streams as well as piloting sensor response in a variety of representative environments and conditions. This study is not intended to examine differences in device behavior between participants, age groups or between male and female subjects. The included exemplar results demonstrate analyses performed to assess the nPS device performance. Irrespective of the test phase, participants for HSR needed to be between the age of 18–60 years old, exercise for at least 60 min at moderate intensity at least 1 day/week, and must be able to walk and swim on their own without any type of aid such as crutches. Exclusion criteria can be found in the Supplementary Appendix.

#### 3.1.1 Dry, normobaric testing

The objective of dry, normobaric testing was to evaluate the baseline performance of the nPS before it was subjected to wet or hyperbaric environments. This initial assessment involved three main test subphases, including: 1. vaso-occlusive testing, 2. exercise circuit testing, and 3. device placement testing. The same participants completed all three test sub-phases.

##### 3.1.1.1 Vaso-occlusive testing

This phase of testing aimed to simulate physiological conditions characterized by vasoconstriction at hyperbaric pressures to evaluate sensor agreement in measuring blood and tissue oxygenation in such environments. During vaso-occlusive testing, the nPS and Moxy Monitor quantified changes in regional muscle oxygenation induced by externally applied pressure to restrict blood flow (Chung et al., 2020; Abel et al., 2014; Hyttel-Sorensen et al., 2014). A blood pressure cuff was used to induce graded occlusion, and StO_2_ responses measured by the nPS were compared with those from the Moxy Monitor, a commercially available device that measures skeletal muscle oxygen saturation (SmO_2_).

**FIGURE 2:**
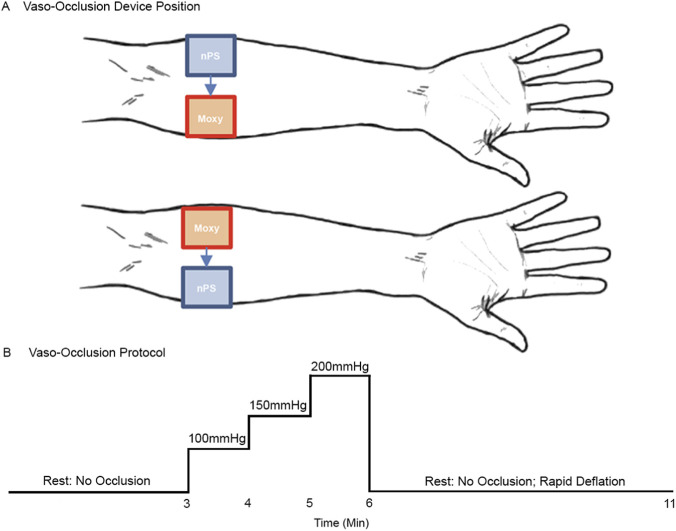
Vaso-occlusive device position and protocol. **(A)** Four occlusion ramps were conducted per participant, with the nPS and Moxy alternating between medial and lateral positions on the volar forearm. **(B)** The protocol progressed through a series of pressures: baseline (no occlusion), 100 mmHg, 150 mmHg, 200 mmHg, and recovery (no occlusion).

Devices were positioned 5 cm apart—the minimum distance to prevent optical signal cross-contamination—on the proximal volar forearm of the dominant hand. Occlusion was repeated with each device placed in both the medial and lateral positions, and average values were compared across locations (Figure 2A). Subjects were seated upright, their arm remained at heart level, and sensors were positioned below the antecubital fossa, specifically 1/3 of the distance from the medial epicondyle of the elbow to the wrist. The devices were aligned with the emitters oriented towards the elbow, and outlines were marked on the skin to ensure consistent placement. Each participant completed four occlusion ramps to counterbalance device (nPS A/B) and placement location on the arm (Medial/Lateral).

The cuff was placed approximately one inch above the antecubital fossa, with the tubing oriented anteriorly. Participant were instructed to inform the researcher of any discomfort, at which point pressure was reduced. Occlusion pressures were chosen to induce graded blood flow restriction in the extremity. Each vaso-occlusive ramp consisted of: no occlusion (3 min), 100 mmHg (1 min), 150 mmHg (1 min), 200 mmHg (1 min), and a final no occlusion period (5 min), depicted in Figure 2B. Devices were then repositioned and the protocol was repeated.

##### 3.1.1.2 Exercise testing

**FIGURE 3:**
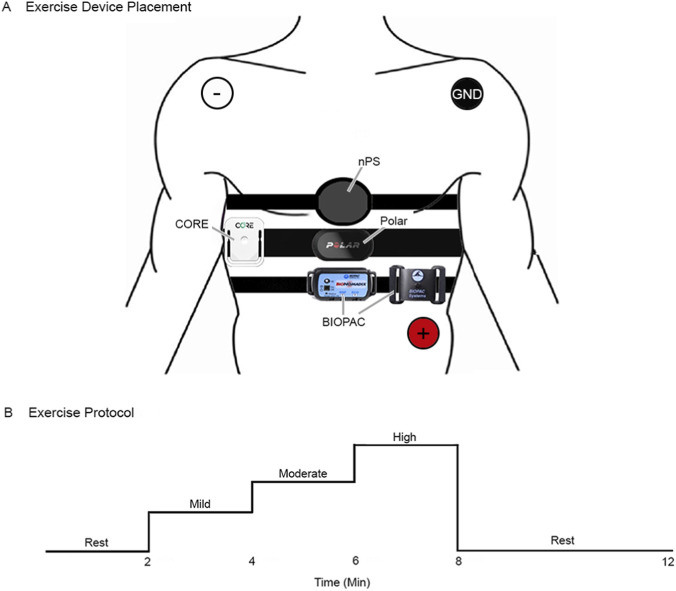
Dry normobaric exercise device placement and protocol. **(A)** Anatomical placement of the nPS, CORE, Polar, and BIOPAC respiration sensor and 3-lead ECG (white: negative, red: positive, black: ground). **(B)** The protocol progressed through a series of exercise intensities: baseline (at rest), mild (50%–60% HR_max_), moderate (60%–70% HR_max_), high (70%–80% HR_max_), and recovery (at rest).

The objective of the exercise circuit protocol was to assess the agreement of the nPS with reference instruments across a range of exercise intensities corresponding to 50%–80% of each participant’s age-predicted maximum heart rate (HR_max_). Participants were equipped with physiological sensing devices (Figure 3A), which included a reference device (BIOPAC), COTS devices (CORE, Polar), and the nPS.

The exercise protocol started with a warm-up stage, where each participant established a comfortable number of revolutions per minute (RPM) on a recumbent bike. This RPM was sustained throughout the test, while resistance levels were adjusted to achieve the required HR zones. The ramped exercise protocol consisted of five stages intended to capture a dynamic range of increasing/decreasing heart rates (Figure 3B): 1. 2 min at rest to acquire baseline metrics, 2. 2 min of mild exercise with the recumbent bike to achieve approximately 50%–60% of the participant’s HR_max_, 3. 2 min of moderate exercise with the recumbent bike to achieve approximately 60%–70% of the participant’s HR_max_, 4. 2 min of intense exercise with the recumbent bike to achieve approximately 70%–80% of the participant’s HR_max_, followed by 5. 4 mins at rest to observe the decline in heart rate back towards their resting HR. After each circuit, participants were prompted to rest until their heart rate returned to within ±10% of their resting heart rate.

##### 3.1.1.3 Device placement testing

The objective of device placement testing was to evaluate and compare the optical signal quality of the nPS at a series of anatomical locations. This assessment intended to identify potential sensing and form factor limitations associated with sensor placement. While the nPS is intended to be worn on the chest, specifically on the sternum, further evaluation of signal quality at various locations along the chest and abdomen was conducted to assess the feasibility of alternative placements. The device placement test protocol took place as part of dry-land testing, but occurred in a separate session from the exercise and vaso-occlusive testing protocols. At the time of this work, the detailed physiological and design rationale for selecting the chest site is still under development by the device manufacturer and is not yet available for disclosure. It has been previously hypothesized, that NIRS measurements from the chest wall may reflect respiratory muscle oxygenation demand, however more evidence needs to be collected (Contreras-Briceño et al., 2019; Espinosa-Ramírez et al., 2023). In this work, the tissue oxygenation measurements recorded should be interpreted to represent local, superficial tissue oxygenation rather than surrogates for other measures.

**FIGURE 4:**
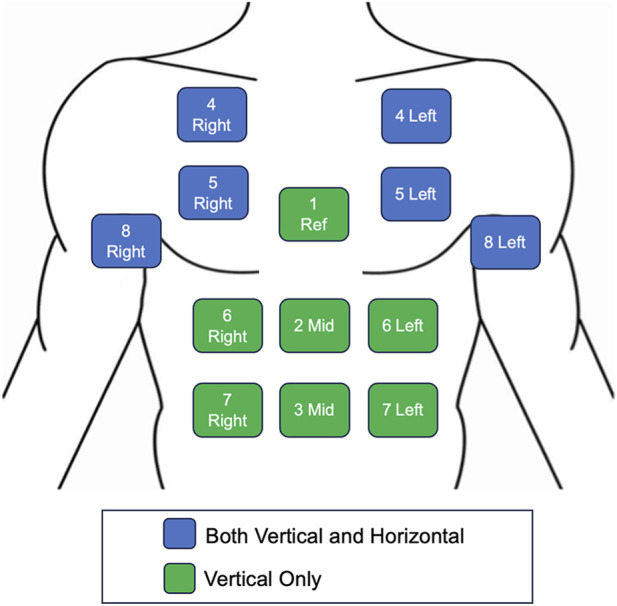
Device placement testing recording locations. Anatomical placement of the nPS along the chest and abdominal regions to assess optical signal quality. The device was position either vertically (shown in green) or both vertically and horizontally (with the source positioned lateral to the detector; shown in blue) at a given site.

The nPS was evaluated at thirteen locations across the chest and abdominal regions (Figure 4). Placement sites included the superior pectoral muscle, the sternum, the abdominal wall, and the axillary regions. To ensure consistent and anatomically proportional positioning across participants, the following standardized measurements relative to each individual’s anatomical landmarks were used:

- Midline Positions:
  o Position 1: One inch inferior to the sternoclavicular (SC) joint
  o Position 2: Two inches inferior to the xiphoid process
  o Position 3: Three inches superior to the navel
- Lateral Positions:
  o Position 4 (Left/Right): Bilateral placements half the distance between each sternoclavicular (SC) joint and deltoid
  o Position 5 (Left/Right): Bilateral placements half the distance between the sternum and each axilla
  o Position 6 (Left/Right): Bilateral placements three and a half inches lateral to position 2
  o Position 7 (Left/Right): Bilateral placements three and a half inches lateral to position 3
  o Position 8 (Left/Right): Bilateral placements two inches inferior to each axilla

The nPS was also tested for two orientations: vertical, in which the sensor’s optical source was positioned superior to its detector, and horizontal, in which the source was positioned lateral to the detector. Testing both orientations was intended to account for potential differences in NIRS-derived signals due to variations in the orientation of underlying skeletal muscle fibers (Sanni and McCully, 2019). The nPS was placed in both orientations at the pectoral and axillar locations (position 4, 5, and 8) and in the vertical orientation only for midline and abdominal locations (positions 1, 2, 3, 6, and 7).

Each participant completed two device placement test cycles—one for each of the two available nPS prototypes. In each test cycle, one device was fixed at position 1 (sternum) as a reference location, while the second device moved through positions 2 through 8 in a randomized order. The sternum was chosen as the reference site based on manufacturer recommendations. Participants were seated upright during testing, and signals were recorded for 30 s at each position. After a test cycle was completed, the procedure was repeated with the two nPS devices switching roles.

#### 3.1.2 Dry, hyperbaric testing

The objective of dry, hyperbaric testing was to evaluate the agreement of the nPS to reference instruments under increased ambient pressures, independent of confounding effects introduced by water immersion. This phase of testing was conducted over multiple days at the UMMC Shock Trauma Hyperbaric Chamber. On each testing day, the participants completed one of three designated dive protocols, each specifying a particular depth and breathing gas mixture (Table 3). Up to three participants completed the dive profile concurrently.

**TABLE 3:** Dry hyperbaric dive profiles.

#	Depth	Descent time	Bottom time	Ascent time	Breathing gas
1	25 ft (7.6 m)	∼4 min	∼106 min	∼10 min*	100% O_2_
2	25 ft (7.6 m)	∼4 min	∼106 min	∼10 min*	Air
3	60 ft (18.3 m)	∼10 min	∼28 min	∼17 min*	Air

*Inclusive of mandatory 5-min safety stop at 20 feet (approx. 6 m).

During each dive profile, participants completed an exercise protocol and a series of cognitive tests, described in detail in the following sections. All dives adhered strictly to ascent and descent procedures outlined in the US Navy Dive Manual Revision 7 (Naval Sea Systems Command, 2016). Descent rates were fixed at 5–10 ft (1.5–3 m) per minute, depending on the participants’ ability to equalize ear pressure, and ascent rates were fixed at 5 ft (1.5 m) per minute. A mandatory 5-min safety stop pas performed at 20 ft (approximately 6 m) during the ascent, during which the participant breathed 100% O_2_. As UMMC is an active treatment facility, all dive profiles were non-decompression dives to ensure participant safety in the event the hyperbaric chamber needed to be quickly evacuated to accommodate patients. The profile with 100% O_2_ breathing gas was conducted in accordance with clinical guidelines and limits laid out in the US Navy Dive Manual, and consistent with certifications held by participating divers.

##### 3.1.2.1 Exercise testing

**FIGURE 5:**
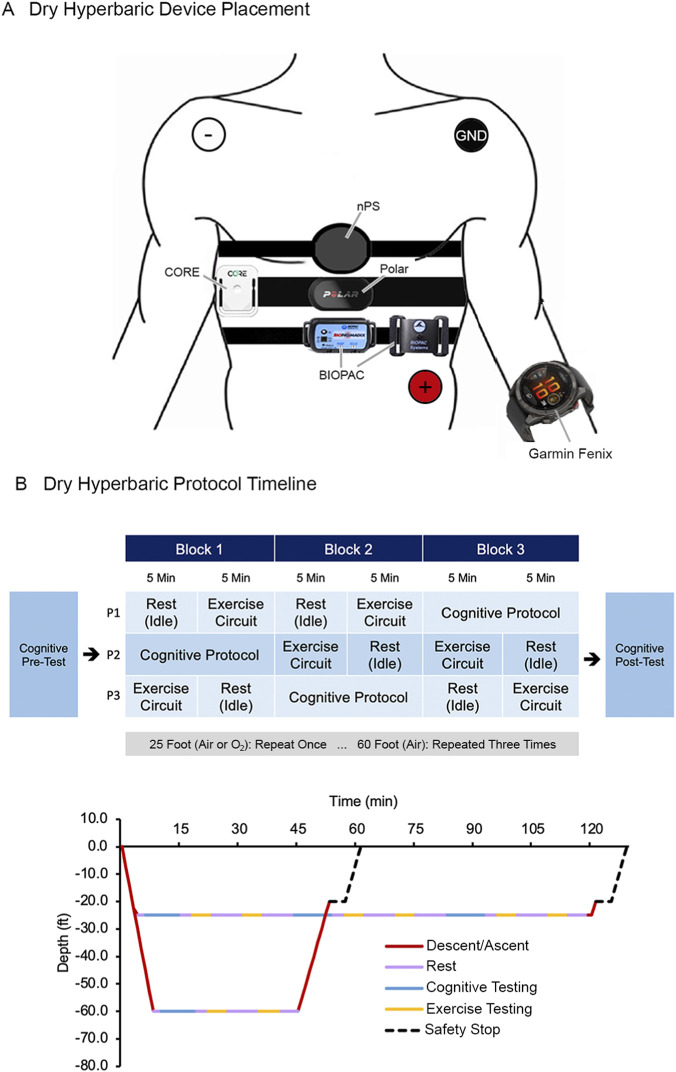
Dry hyperbaric exercise testing device placement and protocol. **(A)** Anatomical placement of the nPS, CORE, Polar, Garmin Fenix, and BIOPAC respiration sensor and 3-lead ECG (white: negative, red: positive, black: ground). **(B)** Timeline for rest, cognitive testing, and exercise testing for 25 ft (7.6 m) and 60 ft (18.3 m) dives.

The objective of the hyperbaric exercise protocol was to evaluate the agreement of the nPS by comparing physiological measurements—HR, SpO_2_, PR—against the BIOPAC reference system and COTS devices (Polar, Nonin, and Garmin Fenix) under elevated ambient pressure (Figure 5A). Physiological metrics were continuously recorded throughout a range of activity intensities. The exercise protocol employed a high-intensity interval training (HIIT) circuit to replicate the dynamic and varied physical demands encountered by divers in operational settings. The HIIT circuit included a series of six 1-min functional movements (Table 4). Participants complete the circuit once during the 60-foot (18.3 m) dive profile and three times for the 25-foot (7.6 m) dive profiles (Figure 5B). No activities were performed during descent or ascent to ensure participant safety and compliance with chamber tender instructions.

**TABLE 4:** Dry hyperbaric HIIT circuit.

Movement	Task	Description
Core/Stability	Trunk Twists	Rotational movement of the torso to engage core muscles for 1 min
Pushing	Alternating Overhead Press	Pressing a 10-pound dumbbell overhead one arm at a time for 1 min
Pulling	Strap Pull Downs	Pulling resistance straps downward to work the back and triceps for 1 min
Simultaneous Leg	Body Weight Squats	Lowering and raising the body using only body weight for 1 min
Independent Leg	Alternating Lunges	Stepping forward into a lunge position alternately with each leg for 1 min

##### 3.1.2.2 Cognitive testing

Although participants remained at depth for up to 2 h, continuous physical activity was not necessary to collect sufficient data for assessing nPS performance. To maintain engagement during this period, a cognitive testing protocol was implemented. Although not directly related to nPS evaluation, this protocol enabled the investigation of adjacent research questions related to diver safety and cognitive performance at depth. Divers must continuously track and manage numerous variables underwater to maintain their own safety. At depth, the underwater environment elicits physiological responses that can alter cognitive functioning while simultaneously increasing cognitive demands. For instance, divers often experience cognitive decline at depth due to factors such as inhaled gas composition (Lafère et al., 2019; Lowe et al., 2007; Grønning and Aarli, 2011), temperature (Lowe et al., 2007; Baddeley et al., 1975; Piispanen et al., 2021; Sharma et al., 2023), pressure (Lafère et al., 2019; Grønning and Aarli, 2011; Brookhuis et al., 1996; Lewis and Baddeley, 1981; Todnem et al., 1989; Dolmierski et al., 1988; Tikkinen et al., 2016), cognitive workload (Todnem et al., 1989; Dolmierski et al., 1988; Er et al., 2017; Stanley and Scott, 1995), and anxiety (Zec et al., 2023; Tsai et al., 2020; Boucher et al., 2022), which can impair attention, memory, and decision-making. When physiological and environmental stressors strain a diver’s cognitive capacity, the likelihood of errors increases, potentially leading to diving-related injuries, disorders, or fatal outcomes. Conducting cognitive assessments in a hyperbaric chamber provides a controlled means of measuring these effects and, when paired with physiological sensors (e.g., EEG, PPG, SpO_2_, HR), enables comprehensive monitoring of diver readiness and performance. Cognitive testing was conducted using an HP EliteBook 840 G11 laptop (Intel® Core™ Ultra 5 135U 16.0 RAM) running Windows (Windows 11 Pro Version 24H2 OS Build 26100.4652).

**FIGURE 6:**
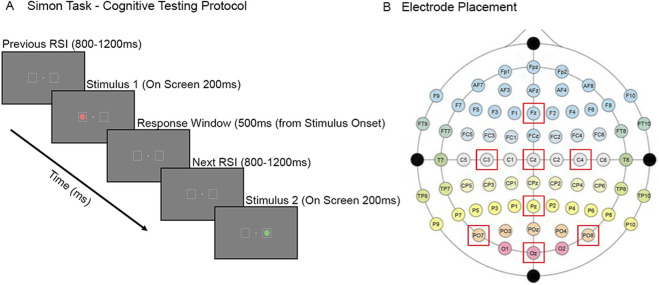
Cognitive paradigm and EEG electrode placement. **(A)** Cognitive test experimental paradigm (Simon Task). **(B)** Electrode placement from the Hybrid Unicorn Black EEG system according to the 10–20 international system.

For the cognitive test, a color-based Simon task was selected to examine neural responses associated with cognitive control, action monitoring, and decision-making (Simon, 1969) (Figure 6A). During the task, participants responded to red or green circles that appeared in one of two boxes on the left/right side of the screen by pressing either the 2 or 8 key on the keyboard (counterbalanced across participants) based on color alone, not screen position. Trials were either congruent (stimulus and correct response mapping were on the same side) or incongruent (stimulus and the correct response mapping were on opposite sides), allowing assessment of error processing and response conflict/inhibition. For a given trial, the stimulus was presented for 200 ms before it was removed from the screen. The participant had 500 ms from stimulus onset to provide a response. Once the response window passed, a jittered response stimulus interval (RSI) of 800 ms–1200 ms was implemented before the next trial started. Responses were still recorded during the RSI window, but responses made after the designated response window were excluded from all analyses. Each block consisted of 160 trials, and participants completed two blocks in a single sitting. Depending on the dive profile, participants completed between six blocks (for 60-foot dives on air) and ten blocks (for 25-foot dives on air or 100% O_2_) per dive.

Participants completed the Simon task before, during, and after each dive, while wearing the Unicorn Hybrid Black (g.tec medical engineering GmbH, Schiedlberg, Austria) (Pontifex and Coffman, 2023), a wireless EEG headset with eight channels (Fz, C3, Cz, C4, Pz, PO7, Oz, and PO8 according to the 10–20 international coordinate system; Figure 6B). The online reference for the device was linked mastoids. There were no channels dedicated to electrooculographic activity, but *post hoc* processing steps were implemented to mitigate artifactual activity due to blinks. The data were collected at a sampling rate native to the device at 250 Hz and were not subjected to any online filtering. EEG event markers were used to align neural data with stimulus presentation and participant responses. This setup allowed for continuous recording of brain activity and *post hoc* extraction of cognitive features empirically correlated with evoked activity in various frequency bands observed in event related spectral perturbation (ERSP) analyses.

Following data acquisition, EEG data were processed using the EEGLAB toolbox (Delorme and Makeig, 2004) in MATLAB (The MathWorks, Natick, MA). Data were detrended and band-pass filtered from 0.1 Hz to 30 Hz using a Butterworth filter (Lopez-Calderon and Luck, 2014). To detect blink-related artifacts—particularly challenging due to the absence of ocular electrodes and the limited number of channels available for independent component analysis (ICA)—a custom pipeline was developed. A duplicate dataset was created in which a peak detection algorithm inserted markers at the peak of each blink. Data were segmented ±500 ms around each blink, and ICA was performed on these short segments, which allowed for reliable identification of the blink component. The resulting ICA weights were then transferred to the original continuous dataset, and the identified blink component was removed. The cleaned continuous data were then segmented into epochs from −1,000 ms to 1,500 ms around all stimulus and response markers. To remove residual artifacts, an automated rejection process was applied using a voltage threshold of 100 µV and a spectral rejection threshold of 50 dB within the 20–40 Hz band (via pop_rejspec) (Delorme and Makeig, 2004). Channels with more than 20% of epochs marked for rejection were excluded and subsequently interpolated using spherical spline interpolation. Participants with more than 10% of channels interpolated were excluded from further analyses. Following preprocessing, the data were transformed into a time-frequency representation using convolution with complex Morlet wavelets at the single-trial level (Cohen and Donner, 2013; Beatty et al., 2020). Frequencies ranged from 2 to 30 Hz in 20 linearly spaced steps, while the number of cycles increased from 3 to 6 in 20 logarithmically spaced steps. Power values were baseline-normalized using a divisive method, relative to the average power from −400 ms to −100 ms relative to stimulus onset. This baseline correction was applied across all conditions of interest using the formula: (10 × log10 (trial N response power/common trial N stimulus baseline period)).

#### 3.1.3 Water immersion testing

The objective of water immersion testing was to evaluate the agreement of the nPS to reference instruments while divers were submerged in a freshwater environment at depths up to 25 ft (7.6 m). Testing was conducted at the NBRF, affiliated with the University of Maryland, College Park. Participant recruitment was conducted by the staff at the NBRF. All participants were certified divers at the facility.

**FIGURE 7:**
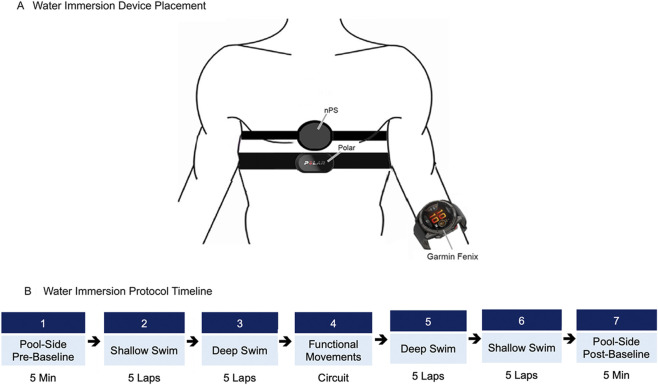
Water immersion device placement and protocol timeline. **(A)** Anatomical placement of the nPS, Polar, and Garmin Fenix. **(B)** Water immersion protocol timeline consisting of pool-side baseline assessment, shallow swim, deep swim, functional movement testing, deep swim, shallow swim, and pool-side post-baseline assessment.

Divers were equipped with the nPS (recording ECG, PPG, StO_2_, SpO_2_, and pressure), the Garmin Fenix (recording PR and pressure), and the Polar Chest Strap (recording HR) as depicted in Figure 7A. Additionally, the Nonin Onyx II was used to measure SpO_2_ during pre- and post-dive poolside baseline recordings. Because Bluetooth Low Energy (BLE) transmission is not possible underwater, data from all sensors were aligned during *post hoc* analysis. To facilitate alignment, all devices were time-synchronized and activated using the ROS 2 system prior to entering the pool. Although signal was lost upon submersion, the system continued to log real-time study events and awaited device reconnection upon resurfacing, aiding in subsequent data alignment. Real-time verbal communication with divers was maintained using underwater communication systems provided by the NBRF.

##### 3.1.3.1 Mock dive scenario

During water immersion testing, participants took part in a “mock dive scenario” that was designed to simulate a range of diver activities (Figure 7B). Prior to submersion, participant physiology was recorded for 5 min to establish a pre-dive poolside baseline. After entering the water, participants were asked to swim 5 laps around the circumference of the pool at the surface before descending to 25 ft (7.6 m) depth and completing another 5 laps (approximately 600 yards or 550 m of swimming). Upon completion, participants performed a range of functional movements representative of common self-contained underwater breath apparatus (SCUBA) diving tasks (Table 5). The goal was to evaluate if the device remained securely positioned and provided accurate readings during diverse motion patterns. All movements were completed sequentially as a circuit, with rest permitted as needed.

**TABLE 5:** Water immersion functional movement testing protocol.

Movement	Task	Description
Pulling	Pull Floatation	Pulling on a rope attached to a floatation device from the surface for 10 reps
Lifting	Kettlebell	Lifting heavy kettlebell/weight overhead from the bottom of the pool (15 Pound kettlebell from the bottom of the pool and lifting it overhead 5 times)
Technical Work	Clip Apparatus	Attaching/Detaching clips from below an underwater apparatus (Simulate divers performing ship maintenance from underneath the ship)
Diving Logistics	Tank Exchange	Changing out the diver’s current oxygen tank for a fresh tank while remaining submerged at the bottom of the pool
Pushing	Dive Brick	Pushing a dive brick/weight for a fixed distance at the bottom of the pool (Length of the pool – from one side to the other)
Navigate Close Quarters	Underwater Vehicle	Getting into/Climbing out of a mock underwater vehicle (Navigate close quarters and squeezing into a confined space.)

Following the functional movement circuit, the participants completed a second round of low-to-moderate exercise, which included swimming 5 laps while at depth, followed by 5 laps just below the surface (approximately 600 yards or 550 m of swimming). After removing diving equipment, participant physiology was recorded poolside for 5 min to obtain a post-dive baseline.

#### 3.1.4 Signal processing

Due to the prototype nature of the nPS and the ongoing, iterative development of the device, some reported features were nonfunctional at the time of evaluation, limiting the scope of analysis for this publication. A key example is skin temperature; although the nPS initially indicated support for skin temperature measurement, this feature was nonfunctional at the time of delivery and was therefore excluded from analysis. Furthermore, although both ECG and PPG signals were acquired by the nPS device, only ECG data were retained for quantitative analysis. PPG signals collected from the nPS were excluded due to system-level acquisition failures that rendered the signal unreliable across multiple test sessions. As a result, all HR derivation and ECG signal quality analyses for the nPS were based exclusively on ECG data.

Heart rate was derived using two complementary approaches, depending on device capabilities. For devices that did not compute HR internally, including the nPS and the BIOPAC reference system, HR was computed offline from recorded ECG signals using a custom Python-based processing pipeline. ECG preprocessing, R-peak detection, and signal cleaning were performed using the NeuroKit2 library (version 0.2.11) (Bizzego et al., 2019), which applies bandpass filtering and artifact correction prior to peak detection. Detected R-peaks were converted to inter-beat intervals and subsequently to instantaneous HR. To reduce beat-to-beat variability and suppress occasional peak-detection jitter, a centered rolling mean over 100 consecutive detected beats was applied to the ECG-derived HR time series.

For commercial devices that reported HR or PR internally—specifically the Polar chest strap, Garmin Fenix smartwatch, and Nonin pulse oximeter—these values were used directly for analysis without additional signal processing. These device-provided metrics reflect proprietary onboard algorithms and were treated as reference or comparison measurements rather than being recomputed from raw signals.

For visualization and population-level comparisons, HR time series were cropped to the experiment duration and segmented according to protocol-defined task phases (e.g., rest, exercise intensity, cognitive task). To enable averaging and paired comparisons across subjects and sessions with variable phase durations, HR data within each task phase were mapped onto a normalized progress axis and resampled to a fixed number of samples (e.g., 500 points) using linear interpolation. This temporal normalization was applied only for population-level time-series averaging and correlation analyses; single-subject plots were generated directly from native timestamped samples without interpolation.

ECG signal quality assessment was applied selectively, depending on protocol objectives. During dry, normobaric exercise testing, ECG-derived HR estimates were used directly for comparison with reference devices, and no formal ECG signal quality analysis was performed beyond the R-peak detection required for HR computation. In contrast, during dry hyperbaric exercise testing and water immersion testing, ECG signal quality was explicitly examined by evaluating R-peak detection behavior across task phases. Using NeuroKit2-based processing, detected R-peak locations and amplitudes were inspected to characterize changes in ECG morphology and the consistency of beat detection over time. This analysis was used to assess the impact of motion, environmental conditions, and sensor displacement on ECG signal integrity and to aid interpretation of agreement between ECG-derived HR estimates and device-reported HR or PR values.

For dry testing protocols (both normobaric and hyperbaric), all physiological signals were time-aligned using the synchronized H5 file generated by the real-time data acquisition system described in Section 2.3. During water immersion testing, where Bluetooth connectivity was unavailable and devices stored data locally, offline temporal alignment was required prior to analysis. In these cases, device-generated timestamps were mapped to the acquisition-system event timeline using logged protocol markers. For ECG-based signals collected by the nPS, cross-correlation of pre-baseline ECG segments was additionally employed when necessary to refine alignment between cached data and the synchronized reference timeline. These alignment procedures ensured consistent temporal correspondence across devices prior to HR derivation, ECG quality assessment, and population-level analyses.

## 4 Results

The following sections describe the pilot results collected across the test procedures described in the methods section. It should be noted that across participants and environments, qualitative human factors questionaries were employed to capture feedback on fit and comfort, affective state, demographics and post-study system usability, among others. These results remain unpublished due to the prototype nature of the nPS, and were primarily utilized by the manufacturer to iterate on the nPS form factor. In all cases, participants did not report adverse impacts on movement or their ability to complete the tasks across the various protocols.

### 4.1 Vaso-occlusive testing results

**FIGURE 8:**
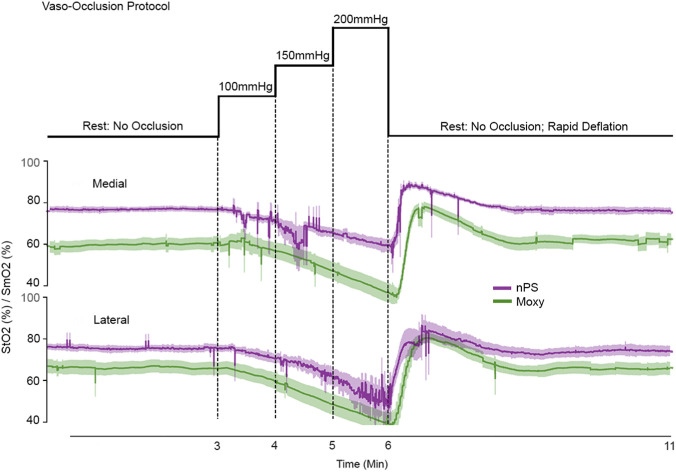
Vaso-occlusive exemplar results. Mean ± SEM of StO_2_ measured by the nPS (purple) and Moxy Monitory (green) across all participants at rest, 100, 150, and 200 mmHg, and recovery.

For vaso-occlusive testing, StO_2_ data were cropped to the experiment duration and normalized across the five occlusion phases (a 3-min pre-occlusion baseline, followed by 1 min each at 100, 150, and 200 mmHg, and a 5-min post-occlusion return to baseline) using cubic interpolation at 1,000 frames per minute. Figure 8 presents data for both the nPS and Moxy muscle monitor at medial and lateral positioning throughout each occlusion protocol, with the nPS largely tracking measurements from the Moxy.

### 4.2 Dry normobaric exercise testing results

**FIGURE 9:**
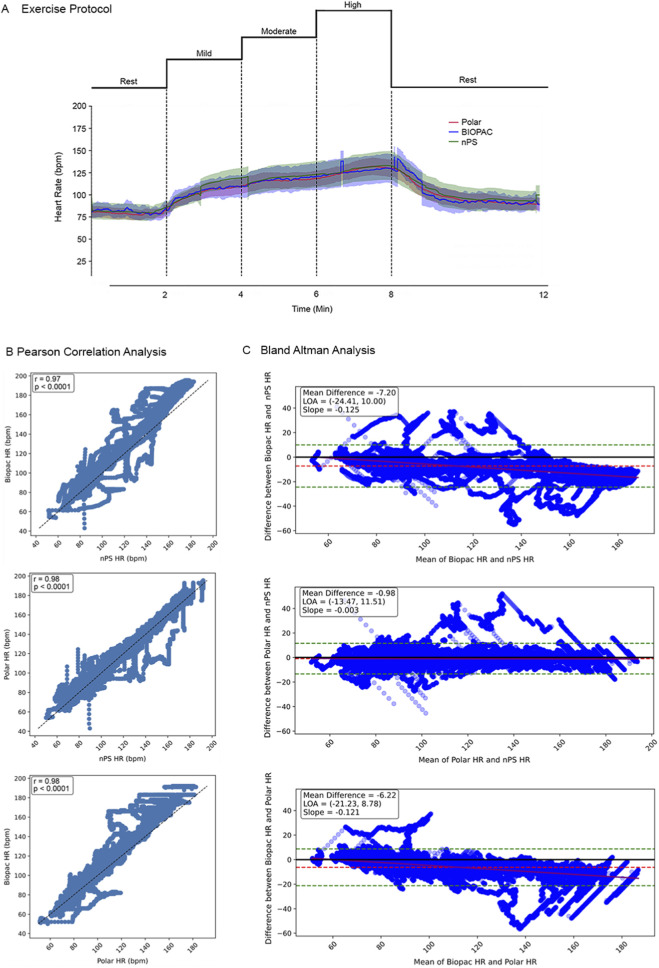
Dry-normobaric exercise testing exemplar results. **(A)** Mean ± SEM of HR measured by the Polar (red) and calculated from ECG from the BIOPAC (blue) and nPS (green) across all participants through the rest, mild, moderate, and high intensity phases of the exercise circuit. **(B)** Pearson correlation analyses comparing HR agreement calculated from BIOPAC, nPS, and Polar. **(C)** Bland Altman analyses comparing HR agreement calculated from BIOPAC, nPS, and Polar.

For dry normobaric exercise testing, HR was derived from ECG data and compared against device-reported HR values following the processing approach described in Section 3.1.4. Figure 9A presents mean ± SEM HR across subjects during the rest, mild, moderate, and high-intensity phases of the exercise protocol. Upon visual inspection, these devices were largely consistent with one another irrespective of exercise intensity. Pearson correlation and Bland-Altman analyses were performed to quantify the agreement of HR data between the nPS, BIOPAC, and Polar. These analyses included 8 participants with 4 experimental repetitions per participant. Experiments with missing or incomplete data were excluded from the analyses. This included Polar data from one participant, as well as five repetitions of nPS data across three participants (due to device failure). Prior to analysis, HR data from all devices were cropped, then down-sampled and linearly interpolated to 10 Hz. Figure 9B presents HR data points compared between BIOPAC/nPS (top), Polar/nPS (middle), and BIOPAC/Polar (bottom) with Pearson Correlation summary statistics in the top left corner. Correlations were significant (p < 0.0001 for all) and showed strong association (r = 0.97, 0.98, and 0.98) for all three comparisons. Figure 9C shows pooled Bland-Altman Analyses, where plots show the difference in HR between devices versus the mean of paired HR values between devices. Upper and lower limits of agreement (LOAs) were calculated as the mean difference ± 1.96 times the standard deviation. Mean HR values measured from BIOPAC showed a small bias (mean differences = 7.20 and 6.22 compared to the nPS and Polar, respectively) and a small, negatively sloped regression line, indicating that values differ more for higher HR values. However, the nPS and Polar showed excellent agreement with a mean difference of 0.98 and a regression line with slope −0.003, indicating little to no change in bias as a function of HR. The data showed clinically significant variance, with LOAs for the three comparisons ranging from −24.41 to 11.51 bpm, which is wider than the typically accepted ideal range of ±10 bpm.

### 4.3 Device placement testing results

**FIGURE 10:**
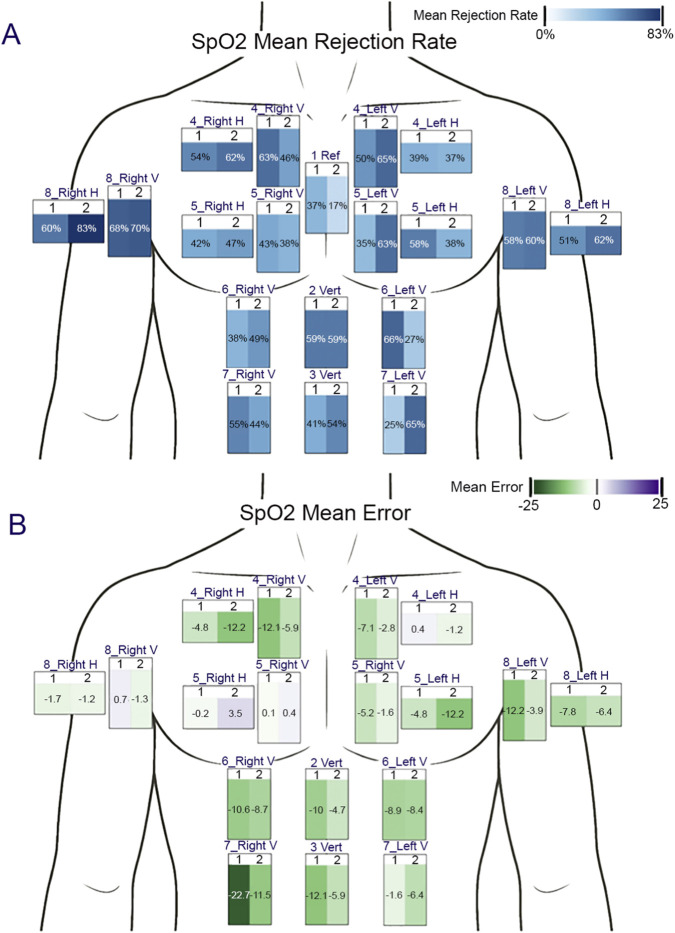
Device placement testing exemplar results. **(A)** Mean rejection rate and **(B)** Mean error rate at each anatomical position on the chest across all participants. The nPS was placed in both vertical and horizontal orientations for pectoral and axillar locations, and only vertically for the midline and abdominal locations.

For device placement testing, StO_2_ values recorded by the nPS at each location were examined for physiological plausibility (e.g., data were rejected if StO_2_ values were below 5% or above 95%, or exhibited abrupt changes greater than 20%). The mean data rejection rate was computed at each test location as a percent of total data recorded across both test cycles and all subjects. The measurement error was calculated as the percent difference between the synchronous StO_2_ values at positions 2 through 8 and those from the reference location (position 1). The mean error rate was then calculated across both test cycles and all participants for each test location. Representative rejection rate and error analyses are presented in Figures 10A,B, respectively.

### 4.4 Hyperbaric exercise testing results

**FIGURE 11:**
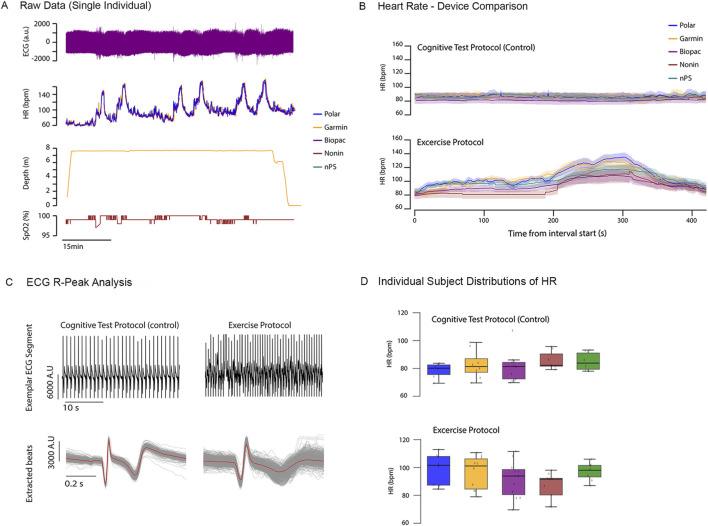
Dry hyperbaric exercise testing exemplar results. **(A)** Exemplar synchronized raw data from the full array of sensors from a single participant. **(B)** Mean ± SEM HR data across all participants during exercise and cognitive phases of testing. **(C)** Sample peak analysis comparing ECG signal quality for a single participant during cognitive and exercise phases of testing. **(D)** Range of HR values observed for individual participants during exercise and cognitive phases of testing.

For dry hyperbaric exercise testing, HR was derived from ECG data and compared against device-reported HR values following the processing approach described in Section 3.1.4. Figure 11A shows representative time-synchronized raw data from all devices for a single participant throughout a dive profile. Figure 11B displays mean ± SEM HR and PR data across subjects during cognitive and exercise phases of testing. In contrast to dry, normobaric exercise testing, ECG signal quality was examined to assess the impact of increased movement and environmental pressure on signal integrity. Representative R-peak analyses of nPS ECG recordings (Figure 11C) demonstrate degradation in ECG morphology and reduced consistency of R-peak detection during exercise phases, which likely contributed to diminished agreement with reference instruments in the derived HR estimates. An additional plot of participant-level HR distributions (Figure 11D) illustrates the range of HR values observed across participants during the exercise circuit, as well as during rest periods associated with cognitive testing.

### 4.5 Hyperbaric cognitive testing results

**FIGURE 12:**
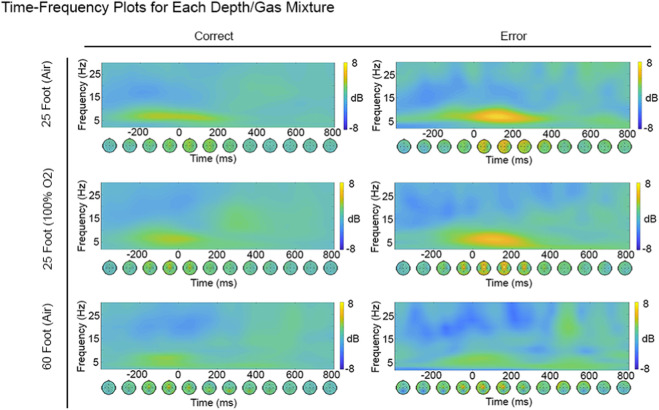
Dry hyperbaric cognitive testing time-frequency plots. Electrophysiological data for correct and error trials (columns) across different dive profiles (rows). In each plot, the y-axis represents frequencies ranging from 1 Hz to 30 Hz, and the x-axis shows time in milliseconds, with time zero aligned to the participant’s response. The color bar indicates power changes in decibels (dB), ranging from −8 dB to +8 dB. Beneath each time-frequency plot is a corresponding topographic time series illustrating the spatial distribution of activity across the scalp.

For hyperbaric cognitive testing, EEG data were processed offline for time-frequency analysis, as described in Section 3.1.2.2. Figure 12 displays electrophysiological data for correct and error trials across different dive profiles. Notably, error trials show a post-response increase in theta power (4–7 Hz), consistent with engagement of the anterior cingulate cortex in error detection processes (Cohen, 2014; Ullsperger et al., 2014; Cavanagh and Frank, 2014). This theta activity is evident in both 25 ft (7.6 m) dives, regardless of gas mixture, but appears reduced in the 60 ft (18.3 m) dive condition. This may suggest a depth-related attenuation of error monitoring processes. However, given the limited sample size, these findings should be interpreted cautiously. Further research is needed to determine whether diving at greater depths impairs cognitive control processes such as error detection—an effect that could have critical safety implications for divers.

### 4.6 Water immersion testing results

**FIGURE 13:**
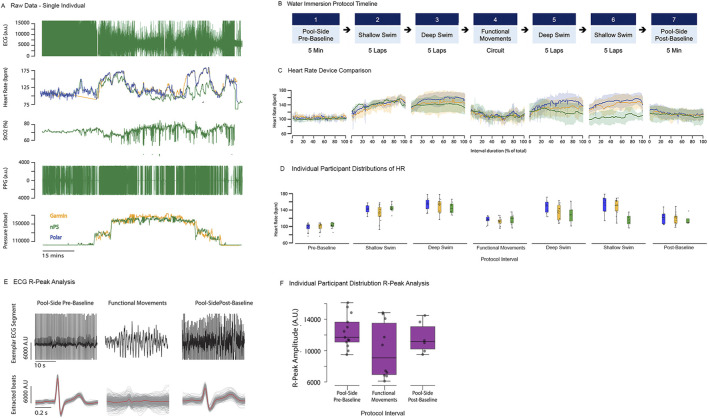
Water immersion testing exemplar results. **(A)** Exemplar synchronized raw data from the nPS (green), Garmin Fenix (yellow), and Polar (blue) from a single participant over the course of a dive. **(B)** Water immersion protocol timeline. **(C)** Mean ± SEM of HR reported by the Polar, Garmin Fenix, and calculated from nPS ECG across all participants for each phase of testing. **(D)** Range of HR values observed for individual participants over the course of a dive. **(E)** Sample peak analysis comparing ECG signal quality for a single participant during the pool-side pre-test baseline, functional movements during the dive, and pool-side post-test baseline phases of testing. **(F)** Individual participant distribution for R-Peak Analysis.

For water immersion testing, HR was derived from nPS ECG data and compared against device-reported HR values following the processing approach described in Section 3.1.4. Figure 13 shows representative time-synchronized raw data from all devices for a single participant throughout water immersion testing. ECG signal quality was explicitly examined during water immersion testing due to increased motion and environmental stressors. R-peak analysis revealed substantial degradation in ECG morphology and reduced consistency of R-peak detection during the dive, particularly during functional movement phases, and to a greater extent than observed during dry, hyperbaric testing. This degradation was likely driven by swelling and displacement of the adhesive patches securing the ECG electrodes, resulting in diminished signal quality and increased variability in ECG-derived HR estimates.

## 5 Discussion

This study presents a methodology for evaluating the performance of a novel physiological sensor relative to research-grade reference instruments and COTS devices under conditions relevant to underwater physiological monitoring. The approach integrates controlled testing across key environmental factors, including water immersion, pressure, and breathing gas composition, to systematically assess how each contributes to measurement variability and device performance. This protocol addresses two major challenges: 1. uncertainty regarding expected physiological responses in underwater environments, and 2. the absence of established gold-standard methods for physiological evaluation under these conditions. Specifically, there is currently no defined set of reference ranges for physiological metrics across varying depths, inspired gas mixtures, or individual fitness levels. Consequently, even if sensors record data underwater, their accuracy in these contexts remains uncertain. This structured and incremental testing framework provides a foundation for future efforts to benchmark physiological monitoring technologies in extreme or operationally relevant environments.

The analyses presented here are intentionally exemplar and are meant to 1. demonstrate the feasibility of the ROS2 Data collection system and 2. to illustrate how key environmental stressors in the diving context (immersion, pressure, and breathing gas composition) affect measurements across devices. This pilot study was not designed or powered to provide generalizable estimates of accuracy (e.g., overall bias or limits of agreement) or reliability metrics suitable for device verification and validation claims. Additionally, given some of the limitations of the prototype nPS, only the Polar H10 and the nPS ECG measurements are directly comparable across all outlined scenarios. While conclusions are limited based on data presented in this study, the prototype nPS displayed strong agreement on HR compared to the BIOPAC and Polar devices during dry-land exercise testing. Conclusive detailed comparison of the device performance under hyperbaric and water immersion conditions were not feasible for this pilot study, due poor data quality resulting from issues with device fitment and data storage failures. Initial analysis of existing data showed degradations in ECG quality resulting from movement on dry-land as well as extended water immersion. These observations will inform subsequent device design improvements to minimize the impact of movement artifacts on data quality and eliminate the impact of electrode swelling underwater. Once a fully functional nPS is built, the study team intends to conduct more exhaustive testing, including evaluation of multiple devices across all scenarios. Given that the protocol prioritized rapid failure discovery over in-depth performance testing, all results should be interpreted as preliminary. While comprehensive accuracy summaries are outside the scope of this pilot, the current study developed a powerful analysis tool that can be reused to design and benchmark future evaluations in similar environments. In the future, a formal verification study will be performed, allowing specific failure modes and reliability metrics to be examined.

The ROS 2 and Docker-based data acquisition system described in this study offers a flexible and scalable solution for time-synchronized, multi-sensor data collection and analysis. The system enabled flexible integration of a range of sensors with varying communication protocols and sampling rates, maintaining temporal alignment and universal timestamps across all data streams. Future studies can leverage this framework to integrate diverse data sources beyond wearable sensors, including environmental monitors, audio and video recordings, screen and keyboard activity, and neurocognitive assessment tools, such as PsychoPy (Peirce, 2009). However, system performance remains constrained by the bandwidth limitations of the host computer’s Bluetooth drivers and available computational resources, which may affect real-time acquisition as the number of connected devices increases. Furthermore, real-time data acquisition during water immersion testing was limited in this pilot study due to impaired BLE transmission. Nonetheless, this framework could be adapted for real-time underwater applications using devices that rely on alternative communication methods (i.e., acoustic or magneto-inductive signaling (Jouhari et al., 2019; Taniguchi et al., 2021)) which transmit through water and could be integrated into the ROS 2 framework.

As a pilot investigation, the sample size of participants was intentionally limited to enable rapid, iterative development of the nPS. However, future studies aiming to apply this protocol for assessing physiological effects or conducting formal verification testing should determine sample sizes based on appropriate statistical power. Power analyses should be performed to estimate the expected impact of environmental factors (such as water immersion, ambient pressure, and breathing gas composition) on signal quality. Pilot data, such as those presented here, can inform these analyses by quantifying within- and between-subject variability under each test condition. Stratified recruitment, or accounting for differences in training status, body composition, or acclimatization, can also reduce confounds and improve the interpretability of device comparisons across conditions.

Future physiological dive monitor verification efforts should adopt an iterative, multi-condition testing framework in which each environmental variable is first isolated and then progressively combined. For instance, the wet and pressurized environmental tests described in this protocol provide insights into the individual effects of water immersion and pressure on physiological signals and device performance. However, integrating these conditions through wet hyperbaric testing (i.e., immersing the device under pressure) could reveal failure modes not apparent in isolated tests, such as water ingress at elevated pressures or reduced sensitivity to physiological signals in high-noise, motion-affected environments. Beyond chlorinated freshwater settings, future work should expand to saltwater environments to evaluate the effects of corrosion on skin-contact signal quality. Progressing from controlled shallow-water tests to open-ocean deployments would further remove environmental constraints, exposing devices to real-world factors such as wave motion, currents, corrosion, and temperature fluctuations.

Testing should also advance toward more physiologically demanding yet controlled conditions. Cold-water immersion, for example, could uncover failure modes in electronics, adhesives, and batteries while enabling measurement of physiological responses such as vasoconstriction and shivering. Because hypothermia poses a major risk for divers (for example: even moderate cold exposure can accelerate onset and necessitate bulkier personal protective equipment) its effects on device placement, form factor, and communication interfaces warrant careful evaluation. The inclusion of vaso-occlusive testing in the current study represents an initial step toward simulating vasoconstriction and assessing its impact on signal quality. Hypoxic testing will also be essential to verify device accuracy under low-oxygen conditions, where increased heart rate and reduced oxygen saturation are critical indicators of early physiological decompensation (Zhang et al., 2014). To prevent potentially fatal hypoxic syncope at depth, a diving monitor should accurately detect SpO_2_ levels below 90%. While technically demanding and safety-sensitive, combining cold-water and hypoxic testing would provide the most realistic simulation of operational physiological stress.

Finally, this methodology could also be extended to assess the cognitive performance of divers in response to environmental and neurological stressors. Integrating physiological monitoring with synchronized behavioral and neurocognitive assessments would enable characterization of how stress, hypoxia, cold exposure, and increased ambient pressure influence both physiological regulation and cognitive function. Coupling these data streams under combined cognitive and environmental load could yield an integrated understanding of human performance, fatigue, and impairment in divers. Such an approach would also support the identification of early biomarkers of cognitive decline or task degradation (i.e., slowed reaction times, increased error rates, or altered heart rate variability) before critical performance thresholds are reached.

## Data Availability

Datasets are available on request subject to prior approval by NMRC/NAMD.
