# Supplementary material for: A methodological framework for validating a multi-domain physiological sensor for divers using a scalable data fusion platform
Source: Front Physiol. 2026 Mar 18;17:1759280. doi: 10.3389/fphys.2026.1759280 (PMC13038580; doi:10.3389/fphys.2026.1759280)
Supplement: Supplementary file 1 [file DataSheet1.pdf]

1 Appendix

1.1 Safety Documentation for Device Testing

1. International Marine Contractors Association (IMCA). *Code of Practice for the Safe Use of Electricity Under Water*. IMCA D 045, R 015.
2. International Marine Contractors Association (IMCA). *Guidelines for Isolation and Intervention: Diver Access to Subsea Systems*. IMCA D 044.
3. International Marine Contractors Association (IMCA). *Use of Battery-Operated Equipment in Hyperbaric Conditions*. IMCA D 041.
4. Naval Sea Systems Command (NAVSEA). *Naval Lithium Battery Safety Program*. NAVSEA INST9310.1c.
5. Naval Sea Systems Command (NAVSEA). *Technical Publication: Navy Lithium Battery Safety Program Responsibilities and Procedures*. NAVSEA S9310-AQ-SAF-010 Rev 3.
6. International Electrotechnical Commission (IEC). *Effects of Current on Human Beings and Livestock – Part 2: Special Aspects*. IEC CEI/TS 60479-2.
7. International Organization for Standardization (ISO). *Medical Devices - Application of Risk Management to Medical Devices*. ISO 14971.
8. International Electrotechnical Commission (IEC). *Medical Electrical Equipment – Part 1: General Requirements for Basic Safety and Essential Performance*. IEC 60601-1.

1.2 Human Subjects Research Exclusion Criteria

- Current injuries that will interfere with ability to safely perform exercise testing.
- Pain when exercising.
- Musculoskeletal disorders affecting gait.
- Use of medications that affect HR, vascular tone, and volume status (beta-blockers, ACE inhibitors, calcium channel blockers, diuretics, etc.).
- High blood pressure needing more than 1 medication for treatment.
- Congestive heart failure.
- Peripheral artery disease with claudication.
- Cancer.
- Pulmonary or renal failure.
- Unstable angina.
- Venous Insufficiency.
- Uncontrolled hypertension (> 190/110 mmHg).
- Orthopedic or pain conditions.
- Abnormal ECG, indwelling pacemaker or cardiac defibrillator.
- Pregnancy.
- Asthma that will interfere with ability to safely perform exercise testing.
- Language or hearing impairment that would significantly limit ability to communicate with the study team.
